# The Influence of Lunar Phases on Complications in Cataract Surgery: An Analysis of 16,965 Patients

**DOI:** 10.1155/2017/1946527

**Published:** 2017-07-16

**Authors:** Eva-Maria Faschinger, Pia Veronika Vécsei-Marlovits, Dieter Franz Rabensteiner, Birgit Weingessel

**Affiliations:** ^1^Department of Ophthalmology, KH Hietzing, Wolkersbergenstrasse 1, 1130 Vienna, Austria; ^2^Karl Landsteiner Institute of Process Optimization and Quality Management in Cataract-Surgery, Wolkersbergenstrasse 1, 1130 Vienna, Austria; ^3^Department of Ophthalmology, Medical University of Graz, Auenbruggerplatz 4, 8036 Graz, Austria

## Abstract

**Purpose:**

Popular beliefs exert an impact of lunar phases on elective surgery. The aim of our study was to evaluate potential correlations between complications in cataract surgery and the phases of the moon during its passage through the zodiac and Fridays that fall on the 13th.

**Methods:**

Patients with complications during cataract surgery were extracted retrospectively from the clinical database from 2010 to 2014. The dates of surgeries were viewed in relation to the phase and the position of the moon (sign of the zodiac).

**Results:**

Of 16,965 cataract surgeries, 132 eyes developed complications. 0.70% developed complications with a waxing moon, and 0.87% with a waning moon (*p* = 0.745). After Bonferroni correction, there were no statistically significant differences between the numbers of complications under the different signs of the zodiac and no complications on Fridays that fell on the 13th.

**Conclusions:**

The analysis of “non-moon-fitting days” for surgery showed quantitative differences, without statistically significant findings. Our results revealed more complications when the moon was waning, which is in contrast to esoteric belief. Patients may be informed that phases of the moon, signs of the zodiac, or a particular date will have no impact on their surgeries.

## 1. Introduction

Lunar effects on health and human behavior have been postulated for centuries [1]. Superstition, moon calendars, and popular beliefs exert a considerable impact on evidence-based medicine, especially on elective surgery [2]. Public interest in the impact of the moon is persistently high. Many individuals believe in correlations or even causality between the course of the moon and their personal lives [3].

In the course of its 29.5-day cycle, the moon passes through its phases. It waxes after new moon and wanes after full moon. In the course of this cycle, the moon also changes roughly every second day from one of the twelve signs of the zodiac to the next. According to esoteric belief, there is a relationship between the phases of the moon and the factors of everyday life such as the quality of sleep and hair growth, as well as the frequency of accidents and surgical complications, seizures, mood disorders, and even a more frequent occurrence of myocardial infarction [4–7]. This may cause patients to request a change in the date of their elective surgery because of the moon being in an unfavorable constellation [7, 8]. Many patients are afraid of the wrong timing of surgery with respect to moon phases and thus influence surgeons as well as other medical staff [9]. According to the moon calendar, surgeries should only be performed on days when the moon is waning because complications are more common and recovery is prolonged when the moon is waxing [8]. For eye surgery, the days when the moon is waxing and in Aries are considered especially unfavorable [8].

In some rural areas, nearly one fifth of the population believes in the impact of moon phases on the outcome of medicine [1]. More than 40% of medical staffs believe that lunar phases affect human behavior [2]. Ten percent of the German population believes in the effects of lunar phases on disease [3, 4]. In a survey conducted in a German hospital, 21.3% of the in-hospital patients had a moon calendar and scheduled their operations according to lunar cycles [10].

Since cataract surgery is one of the most commonly performed procedures and can be timed by the patient, we viewed the frequency of complications following cataract surgery with reference to the phases of the moon and the ruling house (signs of zodiac) on the day of surgery. We also looked for any correlation with Fridays that fall on the 13th of a month.

## 2. Material and Methods

A retrospective, single-center study was performed: Intraoperative complications were retrieved from the records of all cataract surgeries performed at the Department of Ophthalmology, Hietzing Hospital, Vienna, Austria, in the 5-year period from 2010 to 2014. All phacoemulsification were performed by 11 experienced surgeons who had a record of more than 500 operations. However, some members of the surgical team had not been active during the entire 5-year period. Our statistical evaluation of all surgeries showed no difference in the complication rates of the different surgeons.

The analyzed complications included ruptures of the posterior capsule and surgically induced zonulysis with vitreous prolapse requiring vitrectomy. Congenital cataracts in children and combined operations were excluded from the analysis.

The lunar phases for the days in question were taken from moon calendars for Middle Europe. The waxing or waning phase of the moon, the zodiac house it was in, and the time points of complications were correlated to each other. Surgical complications on Fridays that fell on the 13th of a given month were also noted. Every year has at least one and as many as three Fridays that fall on the 13th.

All procedures performed were in accordance with the ethical standards of the institutional and national research committee (EK-17-073-VK), the 1964 Helsinki Declaration, and its subsequent amendments.

Statistics were analyzed with the SPSS Statistics 23 program (IBM, USA). Pearson's chi-square test was used to determine whether the distribution of a dichotomous characteristic (waxing or waning moon; complications yes or no) was identical in both groups. The level of statistical significance was set to *p* < .05. A Bonferroni correction was carried out to neutralize cumulative alpha error due to multiple comparisons.

## 3. Results

**Figure 1:**
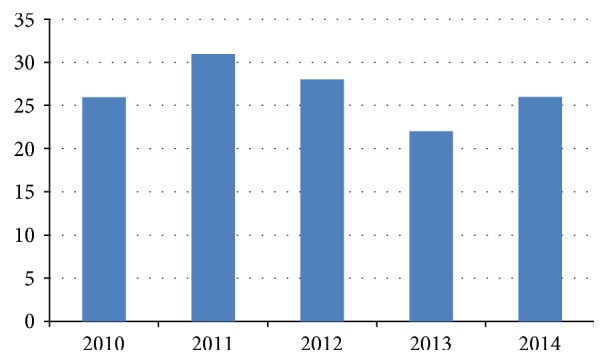
Number of complications during phacoemulsification per year (2010–2014).

The evaluation included 16,965 cataract operations and 132 eyes with complications (0.78%) (see Figure 1 for the individual years).

Complications were somewhat more frequent during a waning moon (*n* = 68) than a waxing moon (*n* = 64). However, 7842 operations were performed during a waning moon and 9123 operations during a waxing moon. In percentages, this amounted to 0.87% complications in a waning moon and 0.70% in a waxing moon (*p* = .745, not significant).

**Figure 2:**
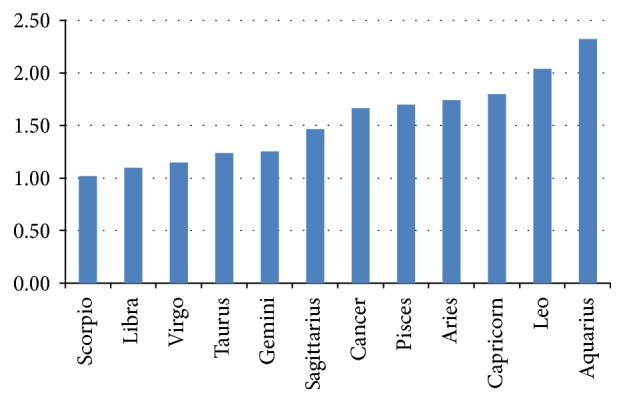
Percent frequency of complications during phacoemulsification per sign (sum of waxing and waning moon, by frequency): least complications in Scorpio, most common in Aquarius.

With regard to the twelve zodiac signs (sum of the waxing and waning moon), complications were most rare in Scorpio (1.01%) and most common in Aquarius (2.32%) (Figure 2). The difference was just slightly nonsignificant (*p* = .051). Listed in chronological sequence, the signs showed the following percentages of complications: Capricorn 1.79, Aquarius 2.32, Pisces 1.69, Aries 1.74, Taurus 1.23, Gemini 1.25, Cancer 1.66, Leo 2.03, Virgo 1.14, Libra 1.09, Scorpio 1.10, and Sagittarius 1.46%.

**Figure 3:**
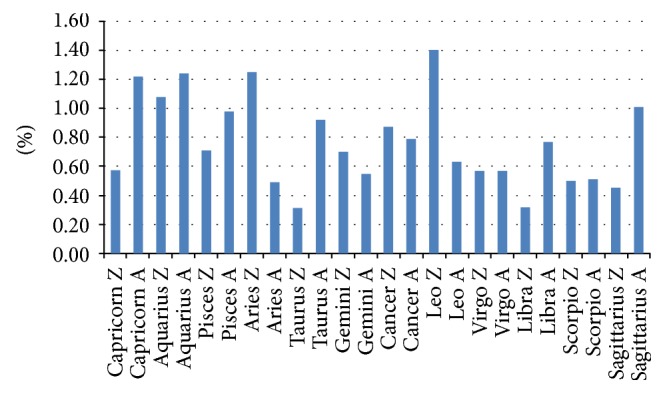
Percent frequency of complications with waxing (Z) and waning (A) moon per zodiac sign: least complications in Taurus (Z, waxing), most common in Leo (Z, waxing).

Viewing the waxing and waning phases of the moon separately, complications were most frequent with a waxing moon in Leo (1.4%) and Aries (1.25%) and with a waning moon in Aquarius (1.24%). The fewest complications were noted with a waxing moon in Taurus (0.31%), Libra (0.32%), and Sagittarius (0.45%) (Figure 3).

Statistically, there were no significant differences between a waxing and a waning moon for the respective zodiac signs (Capricorn *p* = .199, Aquarius *p* = .770, Pisces *p* = .576, Aries *p* = .130, Taurus *p* = .149, Gemini *p* = .737, Cancer *p* = .803, Leo *p* = .162, Virgo *p* = .990, Libra *p* = .269, Scorpio *p* = .993, and Sagittarius *p* = .226).

### 3.1. Subgroup Analyses

A significant difference (*p* = .029) was noted when comparing the first maximal value (Leo, waxing moon) with the first minimum value (Taurus, waxing) as well as with the second minimal value (Libra, waxing, *p* = .035), but no longer with the third minimum value (Sagittarius, waxing *p* = .065). The comparison of the second maximum value (Aries, waxing) with the first minimum value (Taurus, waxing) again yielded a significant difference (*p* = .047), but not with the second minimum value (Libra, waxing, *p* = .056). Comparing the third maximal value (Aquarius, waning) with the first minimum value (Taurus, waxing) yielded no significant result (*p* = .051), nor did a comparison with the second minimum value (Libra, waxing, *p* = .061) (Table 1).

### 3.2. Bonferroni Correction

Twelve zodiac signs and two moon states (waxing and waning) produced 24 groups that, when compared, would lead to 276 possible analogies. Dividing the assumed alpha error of 0.05 by 276, statistical significance was only achieved with *p* < .00018. In these circumstances, none of the results were statistically significant.

During the five-year period of the study, twelve Fridays fell on the 13th of a month (August 2010; January, May, and October 2011; January, April, and July 2012; September and December 2013; and March, June, and November 2014). No complications occurred on Friday the 13th.

## 4. Discussion

Based on the Internet and other publications, many patients request to be operated on days when the moon is “favorable” because they believe that the phases of the moon and the respective signs of the zodiac influence the results of surgery [1–3, 8]. There are many more relevant esoteric Internet sites than there are academic, medical, or natural science-oriented publications, and critical opinions on the subject are few and far between [3, 11].

Our retrospective study on cataract surgeries scheduled in relation to the phases of the moon showed a percentage difference in complications with a waxing moon (0.70%) and a waning moon (0.87%), but the difference was not significant. As the moon passed through the twelve signs of the zodiac, complications were most frequent in Leo, Aries, and Aquarius and least frequent in Taurus, Libra, and Sagittarius. There were no significant differences with the zodiacs and no significant difference depending on whether the moon in the respective house was waxing or waning. Complications within the various zodiac signs were considered in the subgroup analyses. The zodiac sign with the highest number of complications and with the least complications and the phase of the moon (such as a waxing moon in Leo or Aries) revealed a few statistically significant results. However, the results were no longer significant after Bonferroni correction.

Our data showed more complications with a waning moon and do not agree with esoteric beliefs which recommend operations when the moon is waning [8]. The sign of Aries with a waxing moon was associated with the second highest number of complications and was not the most frequent period, as reported in the esoteric literature. We noted the most frequent complications with a waxing moon in Leo. One publication has partly confirmed our data. Gerstmeyer and Lehri checked 8212 surgical reports of phacoemulsification and found no correlations with the lunar phases (waxing and waning). However, they did not take the moon's passage through the zodiac into account, which seems to be important from the patients' point of view [11].

Studies investigating the influence of the moon and its passage through the zodiac are controversial. The majority of them have reported no influence of the lunar phase on survival or complications: Kühnl et al. concluded, from their study in patients with lung cancer, that the moon had no significant effect on intra- and postoperative complications or morbidity and mortality [12]. Wolbank et al. performed a 6-year retrospective investigation of frequency distribution in 11,134 patients presenting for emergency treatment. Clusters were seen (especially for lung diseases), but no relationship was observed between moon phases, the moon's passage through the zodiac, and the number of emergency patients [13]. Another study by Schuld et al. comprising 27,914 emergency cases including blood loss, aortic aneurysm, and gastrointestinal perforations mentions the same conclusion. Besides the moon and zodiac signs, Fridays that fell on the 13th of the month in the nine-year period also showed no accumulation of emergency cases [2]. A retrospective study by Peters-Engl et al. reported no significant difference in survival in breast cancer patients during a waxing (1904 operations) and waning (1853 operations) moon [14]. No significant difference in progression-free survival was noted in 452 patients after radical cystectomy [9]. The very scant literature on the subject also showed no association between surgical complications or medical emergencies and phases of the moon and zodiac signs [13]. A meta-analysis of 37 publications mentioned no connection between lunar phases and “lunatic” behavior (psychiatric disorders and crises, murder, or other criminal behavior) [15]. Lunar phases were not associated with psychiatric admissions or emergency presentation in 8473 psychiatric patients and 1909 emergency psychiatric evaluations [16]. Komann et al. investigated the effect of lunar phases on acute postsurgical pain and treatment-related side effects and assessed datasets of 12,224 patients from 10 international hospitals [3]. The authors disputed the claimed differences in surgical outcomes between lunar phases and concluded that none of the results would justify delaying or skipping surgeries because of moon phases [3].

Some authors report a possible impact: Joswig et al. performed a retrospective analysis of 924 elective spine surgeries and found no influence of unfavorable lunar or zodiac constellations that would justify a moon-calendar-based selection of elective surgery dates, but they also noted that patients undergoing surgery during a waxing moon experienced more intraoperative complications [7]. In a retrospective investigation of 15,985 patients with acute myocardial infarction, Wende et al. observed a possible cardioprotective time of three days after a new moon [17]. The odds of death were reduced at full moon in 210 patients who had undergone repair for dissection in the ascending aorta [18]. A significantly higher number of admissions were noted at full moon in 7219 patients with medically unexplained acute stroke symptoms [1]. A significant clustering of seizures around the full moon period was noted in a review of 859 patients admitted to an emergency unit for seizure [19].

All in all, after cataract operations, we observed nonsignificant differences in intraoperative complications with reference to the phases of the moon (waxing or waning) and its passage through the zodiac. In the subgroup analysis (comparison of the signs with the most numerous complications to those with the least), no significant differences were noted even after Bonferroni correction.

The strength of the present study is the large number of operations. Its weakness is the possible inhomogeneity in the skills of the operating surgeons. No individual quality analyses were performed for the 11 surgeons in respect of the frequency of complications. However, all of the surgeons were experienced. A study with fewer surgeons would possibly provide more conclusive data. We also had no data on the number of risk factors, such as pseudoexfoliation, narrow pupils, or a hard nucleus [20]. Since a surgeon's physical and mental states in terms of his/her concentration abilities, stress, and time constraints vary from day to day, it would be difficult to evaluate these factors. Prospective studies involving one or very few surgeons, with a homogeneous risk profile for the respective eye, would produce more solid results.

Patients who have concerns about a cataract operation planned for an “unfavorable” day with reference to the moon may be informed that there are no conclusive data at the present time to substantiate their concerns. A patient who insists on a more favorable day for the operation can, however, be accommodated when possible.

## Figures and Tables

**Table 1:** Comparison of the three maximum values for complications under the zodiac signs and phase of the moon (Z = waxing, A = waning) to the three minimum values for complications, with reference to the zodiac sign and the phase of the moon. ^∗^Statistically significant in Pearson's chi-square test.

Maximum values	Minimum values	Significance
1st max. Leo Z	1st min. Taurus Z	0.029^∗^
1st max. Leo Z	2nd min. Libra Z	0.035^∗^
1st max. Leo Z	3rd min. Sagittarius A	0.065
2nd max. Aries Z	1st min. Taurus Z	0.047^∗^
2nd max. Aries Z	2nd min. Libra Z	0.056
3rd max. Aquarius A	1st min. Taurus Z	0.051
3rd max. Aquarius A	2nd min. Libra Z	0.061
